# The City and the North: Canada in the Chicago School of Sociology

**DOI:** 10.1007/s12108-025-09657-3

**Published:** 2025-06-02

**Authors:** Sida Liu, Gihad Nasr

**Affiliations:** 1 Faculty of Law, The University of Hong Kong, Hong Kong, China; 2 Department of Sociology, The University of Toronto, Toronto, Canada

## Abstract

This article explores the intertwined histories of the Chicago School of sociology and Canadian sociology, challenging the conventional view that the Chicago School’s development was confined to the city of Chicago. By examining the academic journeys of prominent Canadian scholars such as Annie Marion MacLean, Roderick McKenzie, Helen and Everett Hughes, and Erving Goffman, it illustrates how their contributions were instrumental in shaping both the Chicago School and Canadian sociology. This research, based on extensive archival materials from primary and secondary sources, suggests that the continuous mobility and interaction of scholars between Chicago and Canada played an important role in the historical evolution of sociological thought. The study aims to “decenter” the Chicago School by highlighting the significant yet often overlooked contributions of Canadian sociologists, thereby providing a new understanding of its legacy and global impact.

The history of sociology is closely tied to not only prominent figures but also specific locations. Take the Chicago School, for instance. Most researchers assume that this long-standing sociological tradition was produced exclusively in the city of Chicago since 1892, with generations of ethnographic studies conducted across various neighborhoods but centered around the University of Chicago in Hyde Park (Faris, 1967; Bulmer, 1984; Abbott, 1999; Chapoulie, 2020). This assumption, however, is problematic because it overlooks the diverse life histories of the individuals whose work constitutes the Chicago School. In fact, many major figures of this tradition spent significant portions of their academic careers elsewhere, and few were born and raised in Chicago. Therefore, a comprehensive history of the Chicago School must consider the experiences and contributions of its members in various locations beyond Chicago.

Among these alternative locations, Canada holds a special place in the history of the Chicago School. Annie Marion MacLean, a Canadian woman, was among the first Ph.D. students in sociology at the University of Chicago in the 1890s. Ernest W. Burgess and Roderick D. McKenzie, two key figures of the first Chicago School in the 1920s and 1930s, were both Canadians, as was Erving Goffman in the post-war Second Chicago School. Helen and Everett Hughes, a couple who played a critical role in connecting the first and second Chicago Schools, taught in Canada for more than a decade and contributed to the founding of sociology at McGill University together with Carl Dawson, another Chicago alumnus. Oswald Hall, a student of Dawson as well as Hughes at both McGill and Chicago, played an influential role in post-war Canadian sociology and in garnering respect for sociology at McGill University and the University of Toronto (Clark, 1994; Brown, 2006). It is no exaggeration to say that the histories of the Chicago School and Canadian sociology have been deeply intertwined for over a century.

In this article, we focus on the links between Canada and the Chicago School and present an interconnected history of sociology that is not confined to one or two specific sites but is instead produced by the continuous mobility of scholars and students between “the City” (i.e., Chicago) and “the North” (i.e., Canada). Our theoretical objective is to “decenter” the Chicago School, that is, to move beyond the “metropole” of an intellectual school and demonstrate how this core production site of academic knowledge is made possible through its connections to other locations and, more importantly, the people traveling between them. Some of these connections are intellectual, such as ideas or data traveling across the border, while others are interpersonal or interorganizational, such as the networks formed among scholars that influenced research and teaching. This dynamic applies to both the Chicago School and Canadian sociology– the City and the North found their respective places in the landscape of global sociology through their mutual interactions over time and the mobility of scholars between them.

The archival materials used in this article are drawn from both primary and secondary sources. We benefit greatly from the extensive body of previous research on the history of the Chicago School and the biographies of key figures such as MacLean, McKenzie, Hughes, and Goffman, among other Canadian sociologists. These invaluable studies are complemented by our own primary research conducted at the University of Chicago Library, the University of Toronto Library, and other archives, including the Everett Cherrington Hughes Papers and the Erving Goffman Archives. Additionally, we have meticulously examined the original writings of the scholars discussed in the following pages. Together, these diverse sources provide a good foundation for exploring the interconnected histories and contributions of these Canadian sociologists in the Chicago School.

## The Pioneer: Annie Marion MacLean

The City and the North came into contact long before the Chicago School or Canadian sociology took shape. In 1892, William Rainey Harper, the president of the newly established University of Chicago, appointed Albion Small to set up the first graduate department of sociology in the world. Inspired by the Johns Hopkins model of a research university, Harper recruited Small, who just earned his Ph.D. at Johns Hopkins and became the president of Colby College in 1889. Christian theology had a marked influence on the birth of Chicago Sociology, as Harper was involved in a “sociological movement” to better society through knowledge (Diner, 1975). In addition to Small, Charles R. Henderson, a Baptist minister, was also recruited as a specialist in charity administration in the department. Under Small’s leadership, the sociology department at Chicago grew rapidly in the 1890s and trained the first generation of Ph.D. students in sociology in North America (Abbott, 1999).

Among those early Ph.D. students at Chicago was a Canadian woman, Annie Marion MacLean, who received her master’s degree in 1897 and her Ph.D. in 1900. MacLean was born in 1869 on Prince Edward Island (P.E.I.), Canada. Her grandparents immigrated from Scotland to P.E.I. in 1829 and settled in what was “a forest and no doubt they suffered many hardships and enjoyed few if any of the luxuries of life” (Deegan, 2014: 13). MacLean attended a Baptist-affiliated preparatory school and completed her undergraduate and graduate training at Acadia University in Wolfville, Nova Scotia. She sought to pursue her graduate education at the University of Chicago after having heard about it from her brother, Haddon MacLean. However, after receiving the admission to the sociology Ph.D. program in 1894, Annie Marion MacLean took two years to “garner enough resources, by teaching at Mt. Carrol Seminary in Illinois, before she could begin her doctoral studies” (Forbes, 1900: 303; cited in Deegan, 2014: 3).

Both Annie and Haddon MacLean studied at the University of Chicago in the mid-1890s. Haddon began graduate training in 1892, worked as the secretary of information and exchange in 1896, and became superintendent of the buildings and grounds in 1903 (Deegan, 2014: 25). In 1896, Annie Marion MacLean joined her brother in the university as a full-time student in sociology and studied with Charles R. Henderson, Albion W. Small, George E. Vincent, W. I. Thomas, and Charles Zueblin (Deegan, 2014: 33). George Herbert Mead sat on her master’s committee and, in 1897, she became the first woman to earn her master’s degree in sociology at the University of Chicago (Faris, 1967: 141). Her thesis was titled “Factory Legislation for Women in the United States”, published in the *American Journal of Sociology* in 1897 (MacLean, 1897).

MacLean spent the 1897–1898 academic year collecting data for her Ph.D. thesis in Canada. Her alma mater, Acadia University in Nova Scotia, hosted her and she gave lectures there on “A People within Our Borders, or the Acadians of To-Day” on 14 March 1898 (Deegan, 2014: 33). Based on her fieldwork in Nova Scotia, MacLean completed her thesis titled *The Acadian Element in the Population of Nova Scotia* in 1900. While writing up her Ph.D. thesis, she published another article in the *American Journal of Sociology* on “Factory Legislation for Women in Canada” (MacLean, 1899), a parallel study of her earlier article on the United States. Drawing on statistics from Canada’s seven provinces (British Columbia, Manitoba, New Brunswick, Nova Scotia, Ontario, P.E.I., and Quebec) and the Territories, as well as labor legislations in New Brunswick, Nova Scotia, Ontario, and Quebec, MacLean provided the first sociological analysis of the labor conditions of Canadian women.

Annie Marion MacLean became the second woman to earn a sociology Ph.D. at the University of Chicago. As one of the “new women” of the late nineteenth century “who wanted to bring women into public life and social equality with men” (Deegan, 2014: 2), she was involved in Hull House where feminists such as Jane Addams and Florence Kelley sought to unite ideas and action in a democratic and cooperative community (Schneiderhan, 2011). MacLean adopted this holistic approach to sociology and her studies on factory legislation in Canada and the United States were evidently influenced by Hull House’s agenda for improving labor conditions, especially the work of Florence Kelley. Kelley not only shaped MacLean’s M.A. thesis on factory legislation for women but also provided her opportunities to study labor conditions in Chicago and New York City (Deegan, 2014). MacLean, Addams, Kelley, and other Hull House feminists “formed a tightly interwoven network of scholars and activists interested in similar topics, social justice, and what ultimately emerged as the feminist pragmatist welfare state in the United States” (Deegan, 2014: 74).

Furthermore, MacLean is also recognized for the use of participant observation as well as qualitative, experiential, and quantitative methodologies. She is deemed “the mother of ethnography” by Hallett and Jeffers (2008) for having introduced ethnographic methodologies to the discipline. However, Deegan et al. (2009: 656) criticize this view for failing to “fully or correctly” locate “MacLean’s career within the gendered division of sociological labor” and argue that MacLean’s work was greatly influenced by Harriet Martineau, a feminist ethnographer who wrote the first methods handbook and ethnography titled *Society in America*.

It is also worth noting that religious and family ties were critical for MacLean’s engagement with the University of Chicago in its early years. Growing up in a Baptist family, MacLean shared the same religious background with several founders of the University of Chicago and its Sociology Department, including William Rainey Harper, Albion Small, and Charles R. Henderson, as well as the University’s funders, John D. Rockefeller and the American Baptist Education Society. It was this religious network that brought her to Chicago in the first place. In later years, she formed deeper family ties with Small through her brother Haddon. Haddon married Pearle Harris and Pearl’s brother, Hayden Bartlett Harris, married Albion Small’s daughter, Lina, thus making Lina and Haddon in-laws. As phrased by Deegan (2014: 27), “it is significant that the daughter of Annie’s doctoral chair was also the sister-in-law of Annie’s brother.”

MacLean had hoped to return to Canada after her doctoral study, yet her career plan was severely disrupted due to illness. Although she taught at the University of Chicago as extension assistant professor of sociology in the Home-Study Department from 1903 to 1934, MacLean was for many years in poor health. It was her brother Haddon who assumed her medical costs. Despite her illness, MacLean published eight books and numerous articles, including another article in the *American Journal of Sociology* titled “Significance of the Canadian Migration” (MacLean, 1905). In this piece, she presented statistics of Canadian immigrants in the United States and discussed issues of Canada-U.S. relations and the British Canadian identity. To some extent, the article was a self-reflection on her own experience as an immigrant, aspiring to “be one family” with “Canadians at home, Canadians in the United States, and American citizens” (MacLean, 1905: 823).

As a pioneer in Canadians’ journey from the North to the City, MacLean engaged with not merely one Chicago School of sociology, but multiple Chicago Schools in the making. Her mentors included not only the founders of the Department of Sociology such as Small, Mead, and Thomas but also Jane Addams and other women scholars in the Hull House. Unlike later generations of Canadians in Chicago whose scholarship clearly bore the marks of a particular style of sociology, MacLean’s writings were methodologically plural and theoretically ambiguous. Her intellectual orientation, however, was evidently progressive, arguably a result of her interactions with women and men of the American pragmatists who advocated for the “(re)construction of citizenship, democracy, and peace” (Schneiderhan, 2011: 592).

## The Human Ecologist: Roderick Duncan McKenzie

Two other Canadians soon made significant contributions to the history of the Chicago School. Ernest Watson Burgess, born in Tilbury, Ontario in 1886, moved to the U.S. with his parents in 1888. He pursued graduate studies in the Department of Sociology from 1908 to 1913. In 1916, three years after completing his Ph.D., Burgess returned to the Department as a faculty member, where he remained until his retirement. Despite spending most of his life in the U.S., Burgess retained his Canadian citizenship and did not become a U.S. citizen until later in life. Roderick Duncan McKenzie, born in Carman, Manitoba in 1885, arrived in Chicago in 1913 after completing his studies at the University of Manitoba. While Burgess was Canadian by birth, McKenzie spent the first 28 years of his life in Canada. Therefore, this section will focus more on McKenzie’s contributions to the Chicago School.

Burgess and McKenzie first met at Ohio State University in 1915, where McKenzie was teaching economics and sociology during his doctoral studies, while Burgess served as an assistant professor. After Burgess returned to Chicago in 1916, both collaborated closely with Robert E. Park to develop what would later be known as “human ecology” (Park et al., 1967; Hawley, 1986). Although Park and Burgess received most of the credit for this approach, partly because of their influential textbook, *Introduction to the Science of Sociology* (Park and Burgess [1921], 1969), it was McKenzie who first coined the term “human ecology” in his Ph.D. thesis, *The Neighborhood: A Study of Local Life in the City of Columbus*,* Ohio*, published as five articles in the *American Journal of Sociology* in 1921–1922 (McKenzie, 1923).

It is important to note that human ecology, arguably the signature concept of the first Chicago School, was not only coined by a Canadian but also based on McKenzie’s research in Columbus, Ohio. Although this study was largely “forgotten” by later generations of urban sociologists (Parker, 2020), it challenges the common belief that the Chicago School’s contributions were primarily developed in the city of Chicago through ethnographic work conducted there. In his study, McKenzie examined the community life, disintegration, and social reconstruction of an urban neighborhood in Columbus, as well as the mobility of its population. As Parker (2020) argues, McKenzie’s findings offered the empirical evidence for Burgess’s (1967) concentric zone model, mostly notably in the following paragraph:

“The population of any city is distributed according to economic status into residential areas of various rental of real estate values. Family income tends to segregate the population of a city into different economically districts much the same as the price of tickets at a theatre divides the audiences into several different strata of economic and social distinction.” (McKenzie, 1921: 152).

Additionally, McKenzie investigated the neighborhood as a “unit of social and political reform” (McKenzie, 1922: 780), demonstrating how voter segregation corresponded with the “natural groupings of population” (McKenzie, 1922: 797). These natural groupings, however, were increasingly mobile and transitory, lacking the stability of a traditional community. As he commented in the concluding paragraph of the study:

“However much we may idealize the values of the social solidarity of the traditional neighborhood and long for their return, the fact remains that our social order has changed profoundly from the organic life of the old hamlet or village societies. The seething movements of population show no signs of abating. Community life is ever growing more mobile and transitory.… It is all a phase of the dynamic economic and social order in which we are now living. With the change undoubtedly we lose some of the values which went with solidarity, but, on the other hand, we gain much through the very looseness of the present social structure.” (McKenzie, 1922: 799).

It is challenging to determine the extent to which McKenzie’s perspective on the highly mobile social structure of early 20th-century American society was influenced by his own migration experience from Canada to the U.S. Compared to the rural communities of Manitoba, where he grew up as “the son of Scottish farm parents of venerable lineage” (Hawley, 1968: viii), both Chicago and Columbus represented larger, more transient societies that lacked “organic life” but were full of energy and possibilities. This dynamic and mobile view of society later became a hallmark of the Chicago School’s sociological approach, to which McKenzie made a lasting contribution. It also resonates with Simmel’s depiction of urban life in his seminal work, “The Metropolis and Mental Life” (Simmel, 1971).

In his 1924 article, McKenzie provided the classic definition of human ecology as “the study of the spatial and temporal relations of human beings as affected by the selective, distributive, and accommodative forces of the environment” (McKenzie, 1924: 288). Although the ideas behind this groundbreaking sociological perspective were jointly developed by Park et al. (1967), neither Park nor Burgess formulated definitive statements of human ecology as McKenzie did. As Hawley (1968: xi) commented, “the highly imaginative and restless mind of Park was impatient with details and with the rigors of closely reasoned argument,” while “Burgess, on the other hand, was drawn irresistibly to specific empirical problems.” In contrast, McKenzie not only provided a definition but also systematically outlined the scope of human ecology. This includes ecological distribution, ecological units, mobility and fluidity, ecological distance, ecological factors, and ecological processes (McKenzie, 1968). Ecological processes encompass concentration, specialization, dispersion, centralization, segregation, invasion, and succession, all of which “operate within a more or less rigid structural base” (McKenzie, 1968: 32).

Compared to Burgess’s (1967) classic concentric zone model of urban growth, McKenzie’s ecological approach is arguably more dynamic. While Burgess’s model presents a relatively stable spatial configuration (e.g., the central business district, the transition zone, working-class homes, the commuter zone, etc.), McKenzie focuses on the processes of change within the city and its neighborhoods. For instance, he extensively discussed the processes of invasion, accommodation, and succession in urban development, operating under the assumption that neighborhoods and ethnic groups are always in a state of fluid mobility. This divergence between McKenzie and Burgess, as Hawley (1968: xvi) explains, might be attributed to the fact that “McKenzie, having already embarked upon his teaching career, wrote the dissertation in absentia.” In other words, despite being a core founding member of the Chicago School, McKenzie did not spend much time in Chicago. Although Burgess and McKenzie became “close friends and colleagues on the faculty of Ohio State University” (Hawley, 1968: xvi), their research experiences at the University of Chicago did not coincide.

After earning his Ph.D. in 1920, McKenzie secured a professorship at the University of Washington and spent the next decade in Seattle. During this time, he applied the ecological approach to the Puget Sound region, examining the processes of regional segregation, concentration, integration, and selection, which he described as “the successive stages of regional growth” (McKenzie, 1968: 242). Additionally, he published a commissioned report titled *Oriental Exclusion* (McKenzie, 1927), which addressed the human conditions of Oriental migrants in the U.S. This report “was widely acclaimed for setting the record straight on the contributions made by Orientals to this country and highlighting the harsh effects of exclusion legislation” (Hawley, 1968: x). In the Summer Quarter of 1925, McKenzie returned to the University of Chicago and taught two courses: “The Family” (Sociology 351) and “Human Ecology” (Sociology 361) (University of Chicago, 1925: 161).

In 1930, McKenzie moved to the University of Michigan to chair its newly established Department of Sociology. Until his death in 1940, McKenzie worked on a project to publish a treatise on human ecology. It is believed that the idea for this treatise was “conceived by McKenzie as early as 1924,” and that he agreed to “Park’s request that McKenzie make him coauthor of the book” (MacDonald, 2011: 282). However, “Amos Hawley claimed that McKenzie and Park had a falling out over Park’s alleged use of materials that McKenzie had given him,” which is supported by the fact that there is “no evidence of correspondence” between Park and McKenzie after Park published his article “Human Ecology” in the *American Journal of Sociology* in 1936 (MacDonald, 2011: 282). Eventually, a book on human ecology was completed by McKenzie’s doctoral student Hawley (1950). However, Hawley did not list McKenzie as a coauthor because “McKenzie’s work on the book consisted only of sketchy notes,” and those notes “were lost in a 1950 fire that destroyed the building housing Hawley’s office at the University of Michigan” (MacDonald, 2011: 282).

Therefore, while Park and Burgess built the Chicago School and trained a generation of urban ethnographers at the University of Chicago, McKenzie took a different career path in the states of Ohio, West Virginia, Washington, and Michigan. Despite being a core member of the Chicago School, McKenzie spent little time in Chicago and developed his own research agenda on human ecology. Although McKenzie was often considered a student of Park and Burgess, he was one year older than Burgess and could be regarded as an intellectual peer. Nevertheless, his contributions to sociological theory were overshadowed by Park and Burgess. While human ecology evolved into its own field of study, McKenzie’s name has been largely forgotten by contemporary sociologists.

## The Mid-Century Bridge: Everett Hughes and Helen Hughes

Among Park’s students at the University of Chicago were a couple who met during their graduate studies: Everett Cherrington Hughes and Helen MacGill Hughes. Everett Hughes was born in 1897 in Beaver, Ohio to “a cultivated and widely read Methodist minister” (Riesman, 1983: 477). Everett and those who knew him associated his inspiration to write and pursue sociology with his upbringing experiences of marginality. His father faced religious and racial intolerance from the Ku Klux Klan for being a “nigger lover” and a doubter of the Bible (Riesman, 1983). In contrast, Helen Hughes was a White Anglo-Saxon Protestant (WASP) woman born and raised in Vancouver by Helen Gregory MacGill, a pioneering feminist and one of the first female judges in Canada. Helen studied economics and German at the University of British Columbia, but it was during her senior year that she developed an interest in sociology (Hughes, 1977). During her final term, she received a fellowship to study in the Carola Woerishoffer School of Economics at Bryn Mawr College, but she gave it up after a visit from Robert E. Park during his survey of race relations in the West Coast (Hughes, 1977). Park convinced Helen to attend the University of Chicago instead: “We are starting a new research program, and you’ll learn more there about the things you are interested in than you will at Bryn Mawr.” (Hughes, 1977: 75) When Helen arrived in Chicago in 1925, she met Everett Hughes, who was pursuing his Ph.D. under Park’s supervision.

In 1927, Helen completed her M.A. thesis, titled “Land Values as an Ecological Factor in the Community of South Chicago” (Hughes, 1927). She was “the only woman among six or seven male students” (Eichler, 2001: 381). This thesis is a quintessential example of Chicago School-style ethnography of the 1920s. It begins with a detailed presentation of the neighborhoods and ethnic groups of South Chicago, followed by an analysis of how land values in each neighborhood fluctuate and diminish as one moves away from the city center. In the concluding chapter, Helen applies Burgess’s concentric zone model and discusses “the organic character of the city” (Hughes, 1927: 99).

In 1927, Helen and Everett married in Vancouver, and Everett Hughes started his first academic position at McGill University in Quebec. The following year, Everett defended his Ph.D. thesis, titled *The Growth of an Institution: The Chicago Real Estate Board* (Hughes, 1931). Helen continued to work toward her Ph.D. under Park’s supervision, alternating between Montreal and Chicago to satisfy the University of Chicago’s residence requirements until she received her degree in 1937 (Hughes, 1977). Her Ph.D. thesis, *News and the Human Interest Story: A Study of Popular Literature* (Hughes, 1940), was published by the University of Chicago Press in 1940.

The years Everett Hughes spent in Quebec were pivotal for his career. The Sociology Department at McGill University was established in 1925 by Carl A. Dawson, a Canadian who initially studied theology at the University of Chicago’s Divinity School before shifting his focus to sociology, aligning himself with the emerging Chicago School, particularly Park and Burgess (Shore, 1985). The arrival of Helen and Everett Hughes in 1927 cemented the dominant influence of the Chicago School on the early development of Canadian sociology. As noted on the McGill Sociology Department’s website, “During the first part of this period (the 1920s and 1930s), the world center of sociology was the University of Chicago.” Dawson chaired the department until 1952, a period during which many sociologists trained at the University of Chicago joined the faculty, including Forrest Laviolette^1^, Aileen Ross^2^, Nathan Keyfitz^3^, William Westley^4^, David Solomon^5^, and Fred Elkin^6^. While Ross, Keyfitz, and Solomon were Canadians who studied at the University of Chicago, Laviolette, Westley, and Elkins were Americans who relocated from Chicago to Montreal after earning their Ph.D. degrees. It is no exaggeration to say that Chicago-trained sociologists played a pivotal role in the establishment of Canadian sociology, with McGill University serving as the epicenter of ideas and research activities until the University of Toronto and other institutions gained prominence in the post-war era (Brym, 2002; Cormier, 2002).

While in Quebec, Everett Hughes made a concerted effort to immerse himself in French Canadian culture. He “was fascinated by French Canadian survival” (Fournier, 2002: 45) and expressed criticisms towards McGill University for having “no opportunity for field observation of the French-Canadian language or culture” (Chapoulie, 1996: 14). He went so far as to settle in a French-speaking neighborhood and hired a French-language tutor to aid in his research (Hughes, 1970). Ultimately, his teaching opportunity at Université Laval afforded him the chance to “[live] French” (Hughes, 1970: 14). The exposure to a multilingual setting inspired Hughes’s interest in the influence of industrialization and urbanization on the ways in which different groups of people came together (Riesman, 1983). He conducted fieldwork on the effects of industrialization in Drummondville, a small Quebec town he referred to as “Cantonville” (Hughes, 1943). This research, conducted jointly with Helen Hughes, culminated in his classic book *French Canada in Transition*, first published by the University of Chicago Press in 1943 (Hughes, 1943). This work is perhaps the second major Chicago School study on Canada, following Annie Marion MacLean’s Ph.D. thesis on Nova Scotia. The influence of Robert E. Park’s ecological approach is evident throughout the book; in fact, Hughes dedicated the book to Park.

Hughes’s analysis of Cantonville encompasses not only the group characteristics and social interactions between the French and English populations but also their religious and occupational differences. This focus reflects his lifelong interest in the sociology of work and occupations, an interest that influenced generations of Chicago School sociologists, including Howard Becker, Eliot Freidson, and Andrew Abbott. After a comprehensive investigation of Cantonville in Part II, Hughes turned his attention to Montreal in Part III, discussing the economy and lifestyle of urban French Canadians in contrast to both rural residents and the English. At times, the chapter on Montreal evokes Simmel’s (1971) seminal essay “The Metropolis and Mental Life.” Together with McKenzie’s thesis discussed earlier, this serves as a clear indication of Simmel’s enduring influence on Chicago School sociologists.

In the concluding chapter, titled “Quebec Seeks a Villain,” Hughes argues that “[g]iven the mentality of defense against alien pressure and influence bred by a century and a half of minority existence, it is but natural that French-Canadian eyes should see the hand of cultural aliens in all their difficulties” (Hughes, 1943: 217). In addition to “the military conquest” and “commercial invasions” of the past, the ongoing industrial revolution “moves masses of people from country to city, upsets the equilibriums of the classes, strikes at the very content and aims of education, and threatens a way of life that has, in the past, given comfort and deep satisfaction to its followers” (Hughes, 1943: 217). This emphasis on the impact of modernity on traditional values and lifestyles echoes McKenzie’s conclusion in his study of Columbus (quoted above). However, compared to McKenzie’s optimistic view of change, Hughes appears more sympathetic to the persistent minority status of French Canadians and the disorganization of their lives during industrialization and urbanization.

Hughes’s research influenced a generation of Quebec sociologists, known as “the Laval (Quebec) School,” whose studies rejected “an ethnocentric nationalism in favour of modernity” and focused on “the antinomy between urban American civilization and the religious and cultural identity of the French” (Fournier, 2002: 46). A leading figure of this school was Jean-Charles Falardeau, “the first French-speaking sociologist in Quebec” and “a student of Park and Burgess at the University of Chicago and the ‘heir’ of E. C. Hughes” (Fournier, 2002: 46). Falardeau’s work “brough the ‘inferiority’ of French Canadians to the fore and provided a rationale for two ‘nationalist’ movements” (Fournier, 2002: 46–47) in Quebec in the 1960s.

In 1938, a year after Helen Hughes received her Ph.D., Everett Hughes was offered a faculty position at the University of Chicago. Consequently, the couple moved from Montreal back to Chicago. Everett Hughes is widely recognized as a key mid-century bridge between the first Chicago School and the sociologists trained at the University of Chicago in the 1940s and 1950s (Chapoulie, 1996), including several notable Canadians, most prominently Erving Goffman. The works of the post-war Second Chicago School are believed to have their origins partly in Hughes’s teaching and research (Chapoulie, 1996). However, Hughes himself rejected the notion of a distinct Chicago School. In a conversation with Herbert Blumer, he stated: “I don’t like the idea of talking about a Chicago School or any other kind of school… go ahead and be a Chicago School if you like.” (Lofland, 1980: 276–277) He also reportedly disliked being considered a follower or member of the Chicago School (Helmes-Hayes, 1998).

During this period, Helen Hughes gave birth to two daughters, in 1938 and 1940. She later became an editor at the *American Journal of Sociology*, starting as an editorial assistant in 1944 at the invitation of Herbert Blumer and eventually becoming the journal’s managing editor from 1955 to 1961. In a later article titled “Maid of All Work or Department Sister-in-Law? The Faculty Wife Employed on Campus,” Helen referred to this period as her “17 gratifying, unpaid years” and described it as “a perfect case of Simmel’s *Kreuzung sozialer Kreise* (the interacting of social circles)” (Hughes, 1973: 767, 771). This was because she was not only the wife of Everett Hughes but also a student of Burgess and a fellow student of Blumer, Wirth, and Hughes, all of whom held the AJS editor position during her tenure. Nonetheless, she also pointed out, “[t]hat this was an outcome of sexism is perfectly clear” (Hughes, 1973: 772).

From 1938 until their retirement, Everett and Helen Hughes maintained close connections to Canada and its emerging discipline of sociology. Even in the 1960s, they still read *The Montreal Star* and attended meetings of the Canadian Learned Societies. In his 1970 essay “Teaching as Fieldwork,” Everett Hughes hinted at the emergence of a distinctive Canadian sociology. After students criticized him for assigning American and British publications, Hughes adapted by engaging in what he termed “Canadian sociology,” a concept he attributed to Carl Dawson. He encouraged students to write reports on their own communities and families (Hughes, 1970: 14). According to Hughes, the subtle differences between American and Canadian sociology were rooted in their respective efforts toward emancipation. In Canada, students focused on emancipation from cultural or religious constraints, while in Chicago, students were “still in search of emancipation” even eleven years after similar fieldwork had been conducted in Canada (Hughes, 1970: 14). Although Hughes does not explicitly discuss a potential Canadian influence on American sociology, he concludes his essay by emphasizing the need for flexible teaching-learning relationships that adapt to the evolving socio-political climate– an approach that may have been inspired by his teaching experiences in Quebec.

## The Crypto-Biographical Writer: Erving Goffman

In the post-war era, one of the most prominent Canadians to study sociology at the University of Chicago was arguably Erving Goffman. Despite the extensive literature on Goffman’s contributions to sociology, his biography remains less explored due to his aversion to self-disclosure (Winkin, 1999; Jacobsen & Kristiansen, 2015). In fact, he once remarked, “Only a schmuck studies his own life.” (Shalin, 2014: 2–3) Nevertheless, as Dmitri N. Shalin contends, “much of Goffman’s writing is crypto-biographical” and “his sociological imagination drew on his personal experience” (Shalin, 2014: 3). A notable example is his book *Asylums: Essays on the Condition of the Social Situation of Mental Patients and Other Inmates* (Goffman, 1961) or his autobiographical essay “The Insanity of Place” (Goffman, 1969), which likely reflects his experience caring for his mentally troubled wife in a Maryland psychiatric facility, although he did not explicitly mention this in the book. Similarly, Goffman’s early life in Canada and his interactions with Everett Hughes significantly influenced his later work as a sociologist. This section will focus on this aspect, especially since there is extensive literature on his later life and work as a middle-class man living in the U.S.

Erving Goffman was born in Manville, Alberta, on June 11, 1922, into a Jewish immigrant family from Ukraine. His father, Max Goffman, “served as a Jewish conscript in the Russian Army” (Jacobsen & Kristiansen, 2015: 12) and, after immigrating from Novokrainka to Winnipeg, made a living by “selling haberdashery and dabbling in the stock market” (Shalin, 2014: 12). During Goffman’s childhood, his mother staged amateur plays, and his sister, Frances Goffman Bay, eventually became a professional actress. Erving Goffman “himself took part in a high school staging of Hamlet” (Shalin, 2014: 4). After graduating from St. John’s Technical High School in 1939 and initially studying chemistry at the University of Manitoba, he worked for the Canadian National Film Board in Ottawa in 1943 before attending the University of Toronto to study sociology in 1944. It is plausible that these early experiences with theater and film influenced his development of dramaturgical concepts in his first book, *The Presentation of Self in Everyday Life* (Goffman, 1959).

However, Goffman did not interrupt his university studies in Winnipeg due to a passion for theater or film. Instead, he sought to avoid being drafted into the army, as his father had been. Being a short Jewish man, he feared becoming “a target for hazing if he were in the army” and thus took a government job in Ottawa (Wrong, 2010; Shalin, 2014: 6). His path to sociology was equally serendipitous. While working at the National Film Board, Goffman met Dennis Wrong, an undergraduate student at the University of Toronto who had a summer job there in 1944. When Goffman mentioned to Wrong that he did not want to return to the University of Manitoba to study philosophy, which he found “too specialized for his taste,” Wrong suggested, “Why not try sociology?” (Wrong, 2010). A year later, in 1945, both Goffman and Wrong graduated from the University of Toronto with a B.A. in sociology and anthropology. They both went on to pursue Ph.D. studies in the U.S., at the University of Chicago and Columbia University, respectively, and eventually became influential Canadian-born American sociologists in the mid-20th century.

In addition to Goffman’s experience with theatrical performances, his early life as a Jewish boy in a small town in Manitoba significantly influenced some of the ideas presented in his book *Stigma: Notes on the Management of Spoiled Identity* (Goffman, 1963). As Shalin (2014: 12) notes, “covering up his past was his general practice, and evading his Jewish roots was something of an obsession.” Growing up in a lower-status Jewish family in a small Canadian town during the 1920s and 1930s was a stigmatizing experience, “where to speak another language was to be suspect of being homosexual” (Hymes [1984] 2000: 628). It is not coincidental that Goffman later developed the concept of “tribal stigma,” referring to the type of stigma “that can be transmitted through lineages and equally contaminate all members of a family” (Goffman, 1963: 4). His marginalized childhood experience, as Shalin (2014) argues, contributed to both his theory of stigma and his early article “On Cooling the Mark Out” (Goffman, 1952), which examines how professional con men and other actors help victims adapt to failure.

It is also important to acknowledge the significant influence of Everett Hughes on Erving Goffman. Goffman’s decision to pursue his Ph.D. at the University of Chicago was made following “an encounter with fellow Canadian sociologist Everett C. Hughes” (Jacobsen & Kristiansen, 2015: 12). Although W. Lloyd Warner primarily supervised Goffman, Hughes also served as a major mentor during his time at Chicago. Goffman enrolled in Hughes’ seminar on work and occupations “where he first heard the expression ‘total institutions’” (Fine & Manning, 2003: 35). In a 1980 interview, Goffman stated, “My teachers were Park, Burgess, and Louis Wirth. And then later on Everett Hughes.” (Verhoeven, 1993: 321)^7^ Interestingly, Goffman rejected the label of “symbolic interactionism” often associated with him, instead describing himself as “a Hughesian urban ethnographer.” Alongside Howard Becker, Joseph Gusfield, Eliot Freidson, and others, he identified that generation of Chicago Ph.D. students as “sociologists of small scale entities like occupations, things like that, with a Hughesian, qualitative, ethnographic perspective” (Verhoeven, 1993: 318–319).

In the same interview, Goffman recalled the intellectual orientation of the University of Chicago in the 1940s: “When I was in Chicago in the 40s, one could still combine lots of different things: Ecology and social organization, class analysis and Warner, and the like.… Everybody read all the articles in the journals, and one took courses across the board, and one didn’t draw those sort of lines” (Verhoeven, 1993: 333) between quantitative and qualitative sociology. He used Hughes as an example of this methodological eclecticism: “But Everett Hughes who stands for Ethnographic Sociology himself used numbers whenever he had the chance. If you look at his book on *French Canada in Transition* [1943], it’s got such quantitative data as he can bring to bear. So that line wasn’t sharp.” (Verhoeven, 1993: 334).

This intriguing connection between Hughes and Goffman is largely overlooked in the extensive literature on the Chicago School. While Hughes is typically regarded as a sociologist of work and occupations, Goffman is often associated with microsociology and symbolic interactionism. However, deep within their intellectual orientations lies a common theme influenced by both scholars’ experiences in the City and the North, which Goffman referred to as Hughesian urban ethnography. Goffman’s significant contribution to this tradition is his exploration of the intimate and sometimes uncomfortable corners of human interactions. Perhaps the best illustration of that is Hughes’s comments on Goffman in his review of *Interaction Ritual: Essays on Face-to-Face Behavior* (Goffman, 1967):

“Goffman and Lorenz work in the same way. They observe with aggressive intensity the behavior of their subjects in situations, or occasions. Observation of this measure of intensity is indeed a threat to the face of the observed subjects, if they are moral beings. Laden with guilty embarrassing knowledge, Goffman saves the face of all who are content to let themselves be considered normal, fallible human beings. Those who cannot take his analysis are no doubt tempted to do away with him and, perhaps, with all sociologists. Those who can take it, will find their minds flooded by free association, with embarrassing memories from their own experience, and with corroborating incidents.” (Hughes, 1969: 426).

## Conclusion

What makes the Chicago School? If we adopt the Chicago School’s interactionist approach to answer this question, we find that this school of thought, like any other, is constituted by the scholars and students who convene and interact in specific locations during particular time periods. However, the city of Chicago and the University of Chicago located in its South Side represent only one of many locations that contributed to the formation and dissemination of the Chicago School of sociology. In this article, we have explored the academic trajectories of several Canadian sociologists who studied at the University of Chicago but spent much of their careers and lives in the North and other places, including Annie Marion MacLean, Roderick McKenzie, Helen and Everett Hughes, and Erving Goffman.

Given more space, our list could easily be extended to include many additional Canadian students who pursued their studies at the University of Chicago, alongside Chicago graduates who ventured north to teach and conduct research in Canada. The experiences of these scholars are diverse, each uniquely shaped by personal circumstances and academic pursuits. It is often challenging to disentangle their “Canadian experiences” from their broader life experiences, as both are integrated into their scholarly identities. Nonetheless, it is through their academic contributions and life journeys that the histories of the Chicago School and Canadian sociology became deeply intertwined throughout the 20th century.

Examining the case of Canada serves as an initial step toward developing a “decentered” history of the Chicago School of sociology– one that moves beyond its traditional “metropole” and emphasizes the movement and interaction of its members across diverse geographical locations and social contexts over time. This decentered history may seem unconventional, yet it is essential for fully recognizing the contributions of those sociologists who passed through Chicago but made their marks elsewhere. Many of these scholars have been forgotten and excluded from the established “canon” of the Chicago School (Faris, 1967; Bulmer, 1984; Abbott, 1999; Chapoulie, 2020). Nevertheless, the intricate web of social circles (Simmel, 1955) they constructed, alongside those who remained in Chicago, has extended far beyond the physical and intellectual boundaries of the City or the North. This fearless spirit of boundary spanning over space and time is perhaps what ultimately defines the Chicago School.

## Data Availability

Not applicable.

## Footnotes

1 Forrest LaViolette (1904–1989) was born in Devil’s Lake, North Dakota, and grew up in Oregon. After receiving his B.A. in anthropology in 1933, he began graduate studies in sociology at the University of Chicago. After completing his Ph.D. dissertation, he accepted a position as an assistant professor of sociology at McGill University in 1940. During the postwar times, LaViolette became a major supporter of Japanese Canadians and their citizenship rights.

2 Aileen D. Ross (1902–1995) was born into a wealthy Montreal family. After earning her B.Sc. from the London School of Economics in 1939, she furthered her education at the University of Chicago, obtaining her M.A. in 1941 and her Ph.D. in sociology in 1951 with a dissertation on Anglo-French relations in Canada. Ross began her academic career during World War II as a Lecturer in Sociology at the University of Toronto (1942–1945). She joined McGill University in 1944 and taught in the Department of Sociology until her retirement in 1970.

3 Nathan Keyfitz (1913–2010) was a pioneer in mathematical demography. During the Great Depression, he earned a B.Sc. in Mathematics from McGill University in 1934, embarking on his professional journey as a research statistician with the Canadian Dominion Bureau of Statistics, where he eventually became a senior statistical advisor. In 1951, he completed his Ph.D. in sociology at the University of Chicago with a dissertation on Canadian fertility. Keyfitz held positions at renowned universities including Toronto, Montreal, Chicago, Berkeley, Harvard, and Ohio State. He taught part-time at McGill University while in Montreal. His works helped shape the field by illustrating how simple mathematical tools can address complex demographic issues.

4 William Anthony Westley (1920–2012) was raised in the New York City area. He pursued studies at Cornell University and the University of Chicago until World War II interrupted his education. Serving in intelligence during the war, Westley later earned his Ph.D. in sociology from the University of Chicago. In 1951, he moved to Montreal to join McGill University, where he devoted his career as a professor, Sociology Department Chair, and Director of the Industrial Relations Centre. Notably, between 1953 and 1957, Westley was an executive officer with the McGill Association of University Teachers, contributing to faculty governance and representation.

5 David Nathan Solomon (1917–1981) was a Canadian sociologist and anthropologist whose scholarly career was closely tied to McGill University. Solomon completed his undergraduate studies with a B.A. in sociology at McGill University in 1939, followed by an M.A. in 1942. He further advanced his academic pursuits by earning a Ph.D. in sociology from the University of Chicago in 1952. After completing his studies, Solomon joined the faculty at McGill University, where he taught sociology and anthropology for more than two decades and served as Chair of the Department of Sociology from 1971 to 1975.

6 Fred Elkin (1918–2011) was born in Atlantic City, New Jersey, and served as a member of the Signal Intelligence Division in the U.S. Army during World War II. Following the war, he embarked on an academic career in sociology that would lead him to significantly influence Canadian social science. Elkin held teaching and research positions at several prestigious Canadian institutions, including McGill University, the University of Montreal, and York University. Throughout his career, Elkin addressed pivotal issues of social change and family dynamics in Canada.

7 Note that Robert E. Park retired from the University of Chicago in 1933 and died in 1944, a year before Goffman completed his undergraduate study at the University of Toronto. Hence, Park’s influence on Goffman was mostly through his publications and his students who later became Goffman’s teachers.

## References

[CR1] Abbott, A. (1999). *Department and discipline: Chicago sociology at one hundred*. University of Chicago Press.

[CR2] Brown, D. M. (2006). Oswald hall, phd: Pioneer Canadian sociologist; 1924–1976. *Journal of the Canadian Chiropractor Association*, *50*(4), 271–281. PMC1839996 17549188

[CR3] Brym, R. J. (2002). Canadian sociology: An introduction to the upper thirteen. *The American Sociologist*, *33*(1), 5–11.

[CR4] Bulmer, M. (1984). *The Chicago school of sociology: Institutionalization, diversity, and the rise of sociological research*. University of Chicago Press.

[CR5] Burgess, E. W. (1967). The growth of the City: An introduction to a research project. *The City: Suggestions for investigation of human behavior in the urban environment* (pp. 47–62). University of Chicago Press.

[CR6] Chapoulie, J. M. (1996). Everett Hughes and the Chicago tradition. *Sociological Theory*, *14*(1), 3–29.

[CR7] Chapoulie, J. M. (2020). *Chicago sociology*. Columbia University Press.

[CR8] Clark, S. D. (1994). Oswald Hall and the sociology of work. In *The sociology of work in Canada* (pp. xv-xxv), ed. Audrey Wipper. Carleton University Press.

[CR9] Cormier, J. J. (2002). Nationalism, activism, and the Canadian sociology and anthropology community, 1967–1985. *The American Sociologist*, *33*(1), 12–26.

[CR12] Deegan, M. J. (2014). *Annie Marion MacLean and the Chicago schools of sociology, 1894–1934*. Routledge.

[CR10] Deegan, M. J., Hill, M. R., & Wortmann, S. L. (2009). Annie Marion Maclean, feminist pragmatist and methodologist. *Journal of Contemporary Ethnography*, *38*(6), 655–665.

[CR11] Diner, S. J. (1975). Department and discipline: The department of sociology at the university of Chicago, 1892–1920. *Minerva*, 514–553.

[CR13] Eichler, M. (2001). Women pioneers in Canadian sociology: The effects of a politics of gender and a politics of knowledge. *Canadian Journal of Sociology*, *26*(3), 375–403.

[CR16] Faris, R. E. (1967). *Chicago sociology, 1920–1932*. University of Chicago Press.

[CR17] Fine, G. A., & Manning, P. (2003). Erving Goffman. *The blackwell companion to major contemporary social theorists* (pp. 34–62). Blackwell Publishing.

[CR14] Forbes, J. F. (1900). New members of the university community. *University Record*, *5*(23 November), 302–303.

[CR15] Fournier, M. (2002). Quebec sociology: A discipline and its object. *The American Sociologist*, *33*(1), 42–54.

[CR18] Goffman, E. (1952). On cooling the mark out: Some aspects of adaptation to failure. *Psychiatry*, *15*(4), 451–463. 13014214 10.1080/00332747.1952.11022896

[CR19] Goffman, E. (1959). *The presentation of self in everyday life*. Anchor Books.

[CR20] Goffman, E. (1961). *Asylums: Essays on the social situation of mental patients and other inmates*. Anchor Books. 10.1192/bjp.bp.113.140442 25587580

[CR21] Goffman, E. (1963). *Stigma: Notes on the management of spoiled identity*. Prentice-Hall.

[CR22] Goffman, E. (1967). *Interaction ritual: Essays in face-to-face behavior*. Aldine Publishing.

[CR23] Goffman, E. (1969). The insanity of place. *Psychiatry*, *32*(4), 357–388. 5349841 10.1080/00332747.1969.11023600

[CR24] Hallett, T., & Jeffers, G. (2008). A long-neglected mother of contemporary ethnography: Annie Marion MacLean and the memory of a method. *Journal of Contemporary Ethnography*, *37*(1), 3–37.

[CR25] Hawley, A. H. (1950). *Human ecology: A theory of community structure*. Ronald Press.

[CR27] Hawley, A. H. (1986). *Human ecology: A theoretical essay*. University of Chicago Press.

[CR26] Hawley, A. H. (1968). Introduction. PP. vii in *On human ecology: Selected writings*. McKenzie, R. D. University of Chicago Press.

[CR28] Helmes-Hayes, R. C. (1998). Everett Hughes: Theorist of the second Chicago school. *International Journal of Politics Culture and Society*, *11*(4), 621–673.

[CR33] Hughes, H. M. (1927). Land Values as an Ecological Factor in the Community of South Chicago. University of Chicago M.A. thesis.

[CR29] Hughes, E. C. (1931). *The growth of an institution: the Chicago Real Estate Board*. University of Chicago Ph.D. thesis.

[CR34] Hughes, H. M. (1940). *News and the human interest story: A study of popular literature*. University of Chicago Ph.D. thesis.

[CR30] Hughes, E. C. (1943). *French Canada in transition*. University of Chicago Press.

[CR31] Hughes, E. C. (1969). Review of Interaction ritual: Essays in face-to-face behavior. *American Journal of Sociology*, *75*(3), 425–426.

[CR32] Hughes, E. C. (1970). Teaching as fieldwork. *The American Sociologist*, *5*(1), 13–18.

[CR35] Hughes, H. M. (1973). Maid of all work or departmental sister-in-law? The faculty wife employed on campus. *American Journal of Sociology*, *78*(4), 767–772.

[CR36] Hughes, H. M. (1977). Wasp/woman/sociologist. *Society*, *14*(5), 69–80.

[CR37] Hymes, D. (2000). On Erving Goffman. In G. A. Fine, P. Manning, & Smith (Eds.), *Erving Goffman* (pp. 48–59). G. W. H. Sage (1984).

[CR38] Jacobsen, M. H., & Kristiansen, S. (2015). *The social thought of erving Goffman*. Sage.

[CR39] Lofland, L. H. (1980). Reminiscences of classic Chicago: The Blumer-Hughes talk. *Urban Life*, *9*(3), 251–281.

[CR40] MacDonald, D. W. (2011). Beyond The group: The implications of Roderick D. McKenzie’s human ecology for reconceptualizing society and The social. *Nature and Culture*, *6*(3), 263–284.

[CR41] MacLean, A. M. (1897). Factory legislation for women in the united States. *American Journal of Sociology*, *3*(2), 183–205.

[CR42] MacLean, A. M. (1899). Factory legislation for women in Canada. *American Journal of Sociology*, *5*(2), 172–181.

[CR43] MacLean, A. M. (1905). Significance of the Canadian migration. *American Journal of Sociology*, *10*(6), 814–823.

[CR44] McKenzie, R. D. (1921). The neighborhood: A study of local life in the City of Columbus, Ohio. I. *American Journal of Sociology*, *27*(2), 145–168.

[CR45] McKenzie, R. D. (1922). The neighborhood: A study of local life in the City of Columbus, Ohio– concluded. *American Journal of Sociology*, *27*(6), 780–799.

[CR46] McKenzie, R. D. (1923). *The neighborhood: a study of local life in the city of Columbus, Ohio*. University of Chicago Ph.D. thesis.

[CR47] McKenzie, R. D. (1924). The ecological approach to the study of the human community. *American Journal of Sociology*, *30*(3), 287–301.

[CR48] McKenzie, R. D. (1927). *Oriental exclusion*. Institute of Pacific Relations.

[CR49] McKenzie, R. D. (1968). *Roderick D. McKenzie on human ecology: Selected writings*. University of Chicago Press.

[CR50] Park, R. E., & Burgess, E. W. (1969). *Introduction to the science of sociology* (1921). University of Chicago Press.

[CR51] Park, R. E., Burgess, E. W., & McKenzie, R. D. (1967). *The City: Suggestions for investigation of human behavior in the urban environment*. University of Chicago Press.

[CR52] Parker, J. N. (2020). Forgetting and remembering the Chicago school of Columbus, Ohio: Roderick D. Mckenzie, neighborhoods and inequality. *Inequalities and the progressive era* (pp. 43–55). Edward Elgar Publishing.

[CR53] Riesman, D. (1983). The legacy of Everett Hughes. *Contemporary Sociology*, *12*(5), 477–481.

[CR54] Schneiderhan, E. (2011). Pragmatism and empirical sociology: The case of Jane addams and Hull-House. *Theory and Society*, *40*(6), 1889–1895.

[CR55] Shalin, D. N. (2014). Interfacing biography, theory and history: The case of erving Goffman. *Symbolic Interaction*, *37*(1), 2–40.

[CR56] Shore, M. (1985). Carl Dawson and the research ideal: The evolution of a Canadian sociologist. *Historical Papers*, *20*(1), 45–73.

[CR57] Simmel, G. (1955). *Conflict and the web of group affiliations*. Free Press.

[CR58] Simmel, G. (1971). *Georg Simmel on individuality and social forms*. University of Chicago Press.

[CR59] University of Chicago. (1925). *Annual register, 1924–1925*. University of Chicago Press.

[CR60] Verhoeven, J. C. (1993). An interview with erving Goffman, 1980. *Research on Language and Social Interaction*, *26*(3), 317–348.

[CR61] Winkin, Y. (1999). Erving Goffman: What a life?– The uneasy making of an intellectual biography. *Goffman and social organization: Studies in a sociological legacy* (pp. 19–41). ed. Smith G. Routledge.

[CR62] Wrong, D. (2010). Dmitri Shalin Interview with Dennis Wrong about Erving Goffman. In Dmitri N. Shalin, *Bios Sociologicus: The Erving Goffman Archives*, 1–53. Available at: https://digitalscholarship.unlv.edu/goffman_archives/76 (last date of access: February 8, 2025).

